# Potential Roles of BMP9 in Liver Fibrosis

**DOI:** 10.3390/ijms151120656

**Published:** 2014-11-11

**Authors:** Jianjun Bi, Shengfang Ge

**Affiliations:** Department of Ophthalmology, Ninth People’s Hospital, Shanghai Jiao Tong University School of Medicine, Shanghai 200011, China; E-Mail: jianjun.bi@yahoo.com

**Keywords:** bone morphogenetic protein 9 (BMP9), activin receptor-like kinase 1 (ALK1), endoglin, inhibitor of differentiation 1 (Id1), hepcidin, Snail, liver fibrosis

## Abstract

Liver fibrosis is a common phenomenon that is associated with several pathologies and is characterized by excessive extracellular matrix deposition that leads to progressive liver dysfunction. Bone morphogenetic protein 9 (BMP9) is the most recently discovered member of the BMP family. BMP9 bound with high affinity to activin receptor-like kinase 1 (ALK1) and endoglin in non-parenchymal liver cells. In addition, BMP9 activated Smad1/Smad5/Smad8 and induced the expression of the target genes inhibitor of differentiation 1 (Id1), hepcidin, Snail and the co-receptor endoglin in liver cells. Although the role of BMP9 in liver fibrosis is currently poorly understood, the presence of BMP9-activated proteins and its target genes have been reported to be associated with liver fibrosis development. This review summarizes the indirect connection between BMP9 and liver fibrosis, with a focus on the BMP9 signaling pathway members ALK1, endoglin, Id1, hepcidin and Snail. The observations on the role of BMP9 in regulating liver fibrosis may help in understanding the pathology mechanisms of liver disease. Furthermore, BMP9 could be served as a potent biomarker and the target of potential therapeutic drugs to treat hepatocytes fibrosis.

## 1. Introduction

Liver fibrosis is a pathological process in which excessive precipitation of diffusive liver extracellular matrix (ECM) occurs as a result of intrahepatic connective tissue dysplasia caused by a variety of pathogenic factors. Many chronic liver diseases, such as viral hepatitis, alcoholic liver disease, fatty liver, and autoimmune diseases, can cause fibrosis [1,2]. In response to various factors that may cause liver damage, liver cells undergo degeneration, necrosis and apoptosis. In combination with liver tissue inflammation, these pathological effects lead to the release of inflammatory cytokines and chemical neurotransmitters. These factors act on hepatic stellate cells (HSCs), inducing the proliferation and activation/transformation of these cells into myofibroblasts, which subsequently synthesize large amounts of ECM components, such as collagen and proteoglycans. In addition, activated HSCs also produce substances that inhibit protease activity, thereby increasing ECM deposition in the liver. As a result of matrix deposition in the liver, fibrosis is formed [3,4,5]. To date, a variety of signal transduction pathways and cytokines have been demonstrated to regulate the development of liver fibrosis, including transforming growth factor-β1 (TGF-β1), bone morphogenetic protein 7 (BMP7), and Smad [6,7,8].

**Figure 1:**
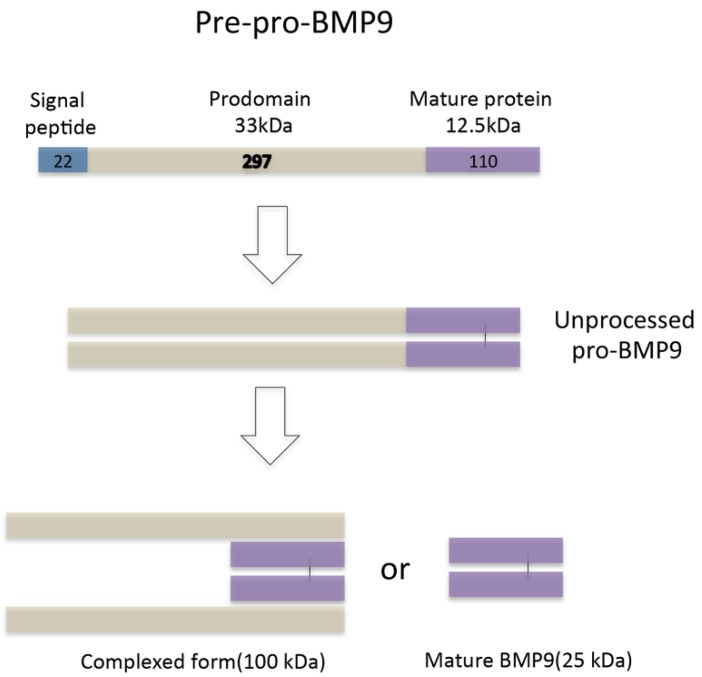
Bone morphogenetic protein 9 (BMP9) biosynthesis. BMP9 is synthesized as a 429 amino acids (aa) precursor protein (Pre-pro-BMP9) composed of a 22 aa signal peptide, a 297 aa prodomain and a 110 aa mature protein. The pre-pro-BMP9 then homodimerizes and is subsequently cleaved to generates two active forms: The short mature BMP9 (25 kDa) and the complexed form (100 kDa).

Bone morphogenetic protein 9 (BMP9), which is also known as growth differentiation factor 2 (GDF2), belongs to the TGF-β superfamily [9,10]. In mammals, the TGF-β superfamily consists of more than 30 ligands, including BMPs, TGF-β subfamily members, activins, seven type I receptors (ALK1-7), and five type II receptors (ActRIIA, ActRIIB, BMPRII, TGF_RII, and AMHRII), as well as several o-receptors and accessory proteins such as endoglin and betaglycan [11]. BMP9 is specifically expressed in liver tissue [12] as a precursor protein (pre-pro-BMP9) composed of 429 amino acids (aa) that include a 22 aa signal peptide, a 33 kDa prodomain and a 12.5 kDa mature protein. The pre-pro-BMP9 is then cleaved by serine endoproteases, leading to a short dimeric mature form of 25 kDa and the prodomain, which can remain noncovalently associated with the mature short form, giving a size of approximately 100 kDa (Figure 1) [13]. BMP9 was initially identified in a murine cDNA library, secreting in both autocrine and paracrine ways [14]. BMP9 regulates several biological functions, including iron ion balance [15], cartilage formation [16], angiogenesis [17,18] and the differentiation of neurons [19], glucose and lipid metabolism [20] When BMP9 acts on liver stellate cells, the regeneration of parenchymal hepatic cells is promoted [12,14].

Little is known about a direct link between BMP9 and liver fibrosis. Fully understanding the relationships between BMP9 signal transduction pathways and multiple target molecules during the fibrotic progression could provide insight into the potential role of BMP9 in liver fibrosis.

## 2. Bone Morphogenetic Protein 9 (BMP9) Receptors and Signal Transduction Pathways in Hepatocytes

Secreted BMPs transduce their signal via a combination of transmembrane serine/threonine kinase receptors, which are comprised of two subtypes: Subtype I (ALK1, ALK2, ALK3 and ALK6) and subtype-II (BMPRII, ActRIIA and ActRIIB). Five membrane BMP9 receptors have been recently reported, these receptors include ALK1, ALK2, BMPR-II, ActRII-B and the co-receptor endoglin [17]. In endothelial cells (ECs), BMP9 binds with high affinity to ALK1 and the co-receptor endoglin. It can also bind to ALK2, BMPRII and ActRII with weak affinity [17,21]. Notably, Townson SA *et al*. reported that BMP9 binds to ALK1 and ActRIIB with high affinity (KD = ~30 pmol) in Biacore experiments, which is significantly higher than those of BMPRII (KD = 2113 pmol) and ActRIIA (KD = 10,370 pmol) [22]. The mode of BMP9–receptor interaction is different from other BMPs and TGF-β members, other BMPs often binding first to the subtype I receptors while members of TGF-β subfamily bind first to the subtype I receptors. These factors activate different signal transduction molecules to mediate the hepatic fibrosis process [23]. The affinity of BMP9 for its receptors on different cell types varies. In cells that do not express ALK1, such as parenchymal hepatic cells, the functions of BMP9 are mediated by the high affinities for ActRII or ALK2 [17,24,25].

Under physiological conditions, BMP9 is primarily expressed and secreted by the liver [12,14,26]. Expression of the BMP9 receptors ALK1, ALK2, ActRII and BMPRII is observed in liver tissue, primary liver cells and liver cancer cell lines [27,28,29,30], indicating that BMP9 may play biological roles in liver cells through these receptors. However, the exact mechanism of action and the biological effects of BMP9 are yet to be studied.

The key aspects of the development of liver fibrosis are the activation of non-parenchymal hepatic cells and the imbalance of the cellular functions. Previous experiments demonstrated that HSCs [31], bile duct ECs and Kupffer cells [14,32] primarily express the type I receptor of ALK1, which can bind to BMP9 and subsequently activate intracellular signaling pathways [24].

**Figure 2:**
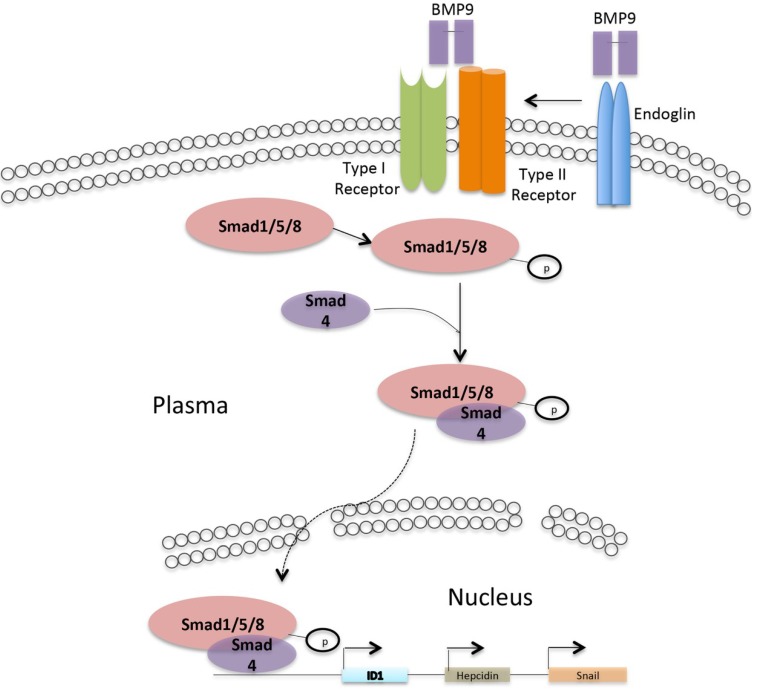
BMP signaling pathway. BMP9 signal transduction in liver cells: BMP9 signal via the type I receptor (ALK1, ALK2) and type II receptor (BMPR2, ActRII) in liver cells. The co-receptor endoglin can modulate signaling via the type II and type I receptors. Activated receptors bind to Smad1, Smad5, Smad8 and phosphorylate the proteins, the activate complex translocates to the nucleus, and bind to Smad-responsive element in the target gene and inducing gene expression.

BMP9 regulates target gene transcription and expression primarily through the Smad pathway [29]. Smad proteins are substrates of BMP receptors such as ALK1. These proteins play an important role in transducing the signal from BMP receptors to target genes within the cell nucleus. The binding of BMP9 to cell membrane receptors induces the formation of a heterologous membrane receptor complex. Activated receptors bind to Smad1, Smad5, Smad8 and phosphorylate these proteins. The activated Smad proteins form a complex, translocate to the nucleus, and bind to a Smad-responsive element in the target gene, eventually inducing the up-regulation of endoglin RNA and the target proteins in ECs (Figure 2) [15,17,31,33].

Other studies have demonstrated that BMP9 can be functioned by phosphorylating Smad2 and Smad3, thus resembling the activation of TGF-β subfamily factors. However, this phenomenon has only been reported to occur in some endothelial cell lines [17,34,35]. No similar observations have been reported in liver cells [24].

Various interactions have been reported between different intracellular signal transduction pathways. Signaling molecules in one pathway may affect or regulate other signaling pathways. One well-known example is the regulation of Smad phosphorylation by the Ras/mitogen-activated protein kinase (MAPK) pathway. In recent years, studies used different protein kinase inhibitors to demonstrate that c-Jun *N*-terminal kinases (JNKs), p38 and ERK1/2 are also involved in BMP9-induced osteogenesis and differentiation of mesenchymal stem cells. The inhibition of MAPK can result in reduced BMP9 function [36,37,38]. Thus, we can postulate that after the Ras/MAPK pathway is activated, activated ERK1/2 can phosphorylate the Smad proteins, regulating the accumulation of these proteins in the nucleus and thereby regulating Smad signal transduction. To date, these non-Smad pathways have rarely been studied in liver cells [24].

## 3. Relationship between Activin Receptor-Like Kinase 1 (ALK1) and Liver Fibrosis

The activation and functional changes of liver non-parenchymal cells are important pathological processes associated with liver fibrosis. The main BMP9 receptor in liver non-parenchymal cells is ALK1 [24,31]. During liver fibrosis, ligand-activated ALK1 activates the target gene Id1 through the Smad1 pathway, thereby inducing HSCs to differentiate into fibroblasts, which produce ECM proteins [39,40]. In other fibrotic diseases, such as scleroderma, research has demonstrated an abnormal overexpression of ALK1 and its associated activated proteins [41,42].

Treating HSCs with the herbal compound 861 (Cpd 861) results in the suppression of ALK1 expression, leading to decreases in key ECM components, such as collagen type III and αSMA, as well as increased expression of matrix metalloproteinase1 (MMP1). Thus, Cpd 861 exhibits anti-fibrotic activity [43,44].

The process epithelial to mesenchymal transition (EMT) leads to pathological changes in tissues. This process occurs in response to specific cytokines. Li *et al*. proposed that EMT may be an important mechanism of liver fibrosis [29,30,45]. They demonstrated that the BMP9/ALK1/Smad1 signaling pathway is involved in the EMT of liver cancer cells. The over-expression of ALK1 leads to up-regulation of the mesenchymal cell marker vimentin and down-regulation of E-cadherin [29].

## 4. Relationship between Endoglin and Liver Fibrosis

Endoglin (CD105) is a co-receptor of TGF-β and BMPs [46,47]. It cannot bind ligand on its own but does bind BMP9 in the presence of type I or type II signaling receptors. Endoglin is a transmembrane protein with large extracellular domains and serine/threonine-rich cytoplasmic regions. Phosphorylation of human endoglin occurs in endothelial cells and mouse fibroblasts. The phosphor-activation status of this co-receptor is regulated by cell surface receptors, including ALK5, ALK1 and Type II receptors, and can also mediate a feedback loop in response to the phosphorylation of ALK5 and type II receptors [48,49]. Endoglin is expressed in ECs, epithelial cells, fibroblasts and HSCs, modulates TGF-β signal transduction by inhibiting the ALK5-Smad2/3 pathway and enhancing the ALK1-Smad1/5 pathway [50,51,52,53].

An immunoprecipitation assay with ^125^I labeled BMP9 and endoglin cDNA-transfected cells revealed a high affinity for BMP9 and endoglin [17]. Endoglin exerts a remarkable regulatory effect on BMP signaling by regulating Smad phosphorylation; it plays an important role in liver fibrosis. Previous experiments demonstrate that during liver fibrosis induced by different causes, the circulating endoglin level is significantly increased. These results suggest that endoglin is involved in hepatitis C virus (HCV)-induced liver fibrosis and can serve as a prognostic biomarker for patients with biliary atresia (BA) liver injury [54,55]. Results reported by Meurer SK *et al.* demonstrate that in murine liver stellate cells, endoglin up-regulates ERK1/2 phosphorylation levels by activating Smad1/5/8. In addition, endoglin increases the expression levels of vimentin, which is an important component of ECM and circulation, and is a connective tissue growth factor [56].

## 5. Relationship between the BMP9 Target Gene Id1 and Liver Fibrosis

Previous experiments using HepG2 liver cell lines and cultured primary cells demonstrate that BMP9 significantly induces Id1 expression [24,57]. As a target gene of BMP9, Id1 plays important roles in the transformation of HSCs into fibroblasts and in the EMT of HSCs [58]. Eliza Wiercinska *et al.* analyze the Smad7-dependent mRNA profile in HSCs cells. They report ectopic Smad7 expression in HSCs with strongly reduced Id1 mRNA and protein expression. They also found that the deletion of Id1 in HSCs impairs the synthesis of αSMA, suggesting that Id1 has a vital function during fibrosis [31]. The results reported by Ding BS *et al.* also suggest that acute injury of sinusoidal ECs induces high expression of the transcription factor Id1, leading to liver regeneration [59]. Matsuda, *et al.* conduct a study with 112 patients and demonstrate that among patients with liver cirrhosis, an increase in Id1 expression is an independent risk factor for the occurrence of hepatocellular carcinoma (HCC). Id1 plays key roles in the early stage of liver cancer development and can be used as a high-risk marker for predicting whether a patient with cirrhosis will eventually develop HCC [60]. A key phenomenon that occurs during hepatic fibrosis is the activation of HSCs to become fibroblasts. In this process, the basic helix-loop-helix (bHLH) transcription factor Id1 plays an important role. The activation of HSCs is accompanied by reduced expression of the inhibitory Id1. The molecular mechanisms that underlie the effects of the Id1 protein on HSC activation and liver fibrosis remain unclear [61].

## 6. Relationship between the BMP9 Target Gene Hepcidin and Liver Fibrosis

In liver cells, another important target gene of BMP9 is hepcidin, which is a cysteine-rich antimicrobial polypeptide. BMP9 can up-regulate hepcidin expression [15]. A significant effect of hepcidin is the inhibition of the absorption and recycling of iron. In the clinic, iron deposition accompanies the hepatic fibrosis and cirrhosis that are caused by a variety of advanced stage diseases [62,63,64]. In patients with chronic hepatitis C, iron deposition in the liver may cause oxidative stress damage and induce apoptosis, thereby contributing to liver fibrosis. Patients with chronic liver disease tend to have disorders related to hepcidin expression and hepatic iron deposition. These effects may ultimately contribute to liver fibrosis [65,66]. Hepcidin expression and the concentration of serum prohepcidin are significantly reduced in patients with chronic hepatitis C. These phenomena are more significant in patients with cirrhosis and are negatively correlated with serum ferritin levels and liver iron content [67,68,69]. Sebastiani G *et al.* used the hemojuvelin Hjv^−/−^ mouse model to study the effects of iron overload on liver fibrosis. They found that the deletion of the Hjv gene leads to the deposition of iron ions and, consequently, promotes liver fibrosis [70]. It is reasonable to postulate that iron deposition in the liver may occur due to the reduction of hepcidin. Thus, hepcidin may be a therapeutic target or a biological marker of iron deposition-associated liver fibrosis. It is possible that hepcidin and BMP9 use different mechanisms to induce liver fibrosis.

## 7. Relationship between the BMP9 Target Gene Snail and Liver Fibrosis

Snail is a key regulator of EMT. BMP9 can induce the expression of Snail in liver cancer cells [29]. When inhibiting Snail-1 activity with the Snail inhibitor, pro-fibrotic genes, such as connective tissue growth factor (CTGF), collagen I and TGF-1, are down-regulated. These effects result in the inhibition of fibrosis and indicate that Snail is involved in fibrosis [71]. Researchers studied the EMT process by inhibiting Snail and reported that inhibiting Snail can inhibit EMT [72]; this finding suggests that the inhibition of Snail can be used to reduce the progression of liver fibrosis.

## 8. Conclusions

Although few studies have investigated the direct correlation between BMP9 and liver fibrosis, the available results demonstrate that BMP9 is primarily expressed in liver cells and acted in both autocrine and paracrine manners. This protein is widely involved in the functions of many receptors linked to liver fibrosis-promoting, the action of downstream signaling molecules and the expression of target genes in liver cells. Our studies and many others postulate that BMP9 is one of factors that may induce liver fibrosis by potentially regulating the process of fibrosis.
